# Molecular Landscapes of Gastric Pre-Neoplastic and Pre-Invasive Lesions

**DOI:** 10.3390/ijms22189950

**Published:** 2021-09-14

**Authors:** Gianluca Businello, Valentina Angerilli, Paola Parente, Stefano Realdon, Edoardo Savarino, Fabio Farinati, Federica Grillo, Alessandro Vanoli, Francesca Galuppini, Silvia Paccagnella, Gianmaria Pennelli, Luca Mastracci, Luca Saragoni, Matteo Fassan

**Affiliations:** 1 Surgical Pathology Unit, Department of Medicine (DIMED), University of Padua, 35121 Padua, Italy; glc.businello@gmail.com (G.B.); valentina.angerilli@gmail.com (V.A.); francesca.galuppini@unipd.it (F.G.); silviapak80@gmail.com (S.P.); gianmaria.pennelli@unipd.it (G.P.); 2 Pathology Unit, Fondazione IRCCS Ospedale Casa Sollievo della Sofferenza, 71013 San Giovanni Rotondo, Italy; paolaparente77@gmail.com; 3 Veneto Institute of Oncology (IOV-IRCCS), 35128 Padua, Italy; stefano.realdon@iov.veneto.it; 4 Division of Gastroenterology, Department of Surgery, Oncology and Gastroenterology, University of Padua, 35121 Padua, Italy; edoardo.savarino@unipd.it (E.S.); fabio.farinati@unipd.it (F.F.); 5 Anatomic Pathology Unit, Department of Surgical Sciences and Integrated Diagnostics (DICS), University of Genova, 16132 Genova, Italy; federica.grillo@unige.it (F.G.); luca.mastracci@unige.it (L.M.); 6 Ospedale Policlinico San Martino, IRCCS for Oncology and Neuroscience, 16132 Genova, Italy; 7 Anatomic Pathology Unit, Department of Molecular Medicine, University of Pavia and Fondazione IRCCS San Matteo Hospital, 27100 Pavia, Italy; alessandro.vanoli@unipv.it; 8 UO Anatomia Patologica, Ospedale G.B. Morgagni-L. Pierantoni, 47121 Forlì, Italy; luca.saragoni@auslromagna.it

**Keywords:** gastric carcinoma, pre-neoplastic lesions, gastric carcinogenesis, biomarkers, pathology

## Abstract

Gastric carcinoma (GC) represents one of the most common and most lethal malignancies worldwide. The histopathological characterization of GC precursor lesions has provided great knowledge about gastric carcinogenesis, with the consequent introduction of effective strategies of primary and secondary prevention. In recent years, a large amount of data about the molecular events in GC development is emerging, flanking the histomorphological descriptions. In this review, we describe the landscape of molecular alterations in gastric pre-invasive lesions with a glance at their potential use in the diagnostic and therapeutic decision-making process.

## 1. Introduction

### 1.1. Epidemiology and Risk Factors

According to the latest GLOBOCAN analysis [1], gastric cancer (GC) is the fifth most diagnosed carcinoma in both males and females, with more than 1 million new diagnoses in 2018. With about 800,000 deaths in 2018, GC also represents the third leading cause of cancer-related death worldwide. Both GC incidence and mortality present substantial differences among countries, with low/middle income areas accounting for more than 70% of cases. The highest GC incidence (up to 32 cases/100,000) is reported in Eastern and Western Asia, Eastern Europe and South America. Conversely, GC incidence in North America and Western Europe has continuously decreased during the last 50 years and is currently standing at less than 7 cases/100,000 [1]. Moreover, in Western countries, a progressive reduction of distal-arising GC has been observed. These trends probably reflect the effects of successful *Helicobacter pylori* (Hp) eradication and improvement of hygiene standards [2]. On the other hand, a higher incidence of cardiac GC has been reported, probably related to increasing rates of gastroesophageal reflux disease and obesity [3]. GC is a multifactorial disease and both genetic and environmental factors are involved in gastric carcinogenesis. GC-associated risk factors include Hp and Epstein–Barr virus (EBV) infection, family history of GC, tobacco smoking, microbiome modifications induced by long-term use of proton-pump inhibitors and high consumption of salt-preserved/smoked foods and of red/processed meat together with low intake of fresh fruit/vegetables [4,5]. In particular, Hp is the environmental factor carrying the greatest weight worldwide, as it is associated not only with GC but also with gastric mucosa-associated lymphoid tissue (MALT) lymphoma [6]. Hp also has a synergistic effect with other risk factors [7]. Inherited GC risk factors are best exemplified by hereditary syndromes with mutations in genes involved in molecular pathways of gastric carcinogenesis. Major syndromes associated with GC are hereditary diffuse gastric cancer (HDGC; mutations in *CDH1*), gastric adenocarcinoma, proximal polyposis of the stomach (GAPPS; YYP1 binding motif of *APC*) and familial intestinal gastric cancer (FIGC) [4,8]. Patients with familial adenomatous polyposis (FAP; *APC*), Peutz–Jeghers syndrome (*STK11*), Li–Fraumeni syndrome (*TP53*) or Lynch syndrome (particularly with *MLH1* or *MSH2* mutations), also have an increased risk of developing GC [9]. Hereditary GCs, related to both cancer susceptibility syndromes and/or other genetic causes, account approximately for 1–3% of cases, and a detailed discussion of these conditions is far beyond the aim of this review [10,11]. Polymorphisms in genes codifying for IL-1beta, TNF-gamma and IL-10 are other GC-related host-dependent genetic factors [12]. GC-related precancerous conditions are chronic atrophic/metaplastic gastritis (AG), pernicious anemia, peptic ulcer disease, previous gastric surgery and Ménétrier’s disease [4,9].

### 1.2. Histological and Molecular Classifications of GC

GC is a molecularly and phenotypically heterogeneous disease. Over the years, numerous morphologic and molecular classifications have been proposed in order to dissect the complexity of this disease. Despite these efforts, a unifying classification of GC is still lacking [13].

The most common histotype of GC, gastric adenocarcinoma (GAC), accounts for more than 90% of GCs [14]. Squamous cell carcinoma, adenosquamous carcinoma, undifferentiated carcinoma, gastroblastoma and neuroendocrine neoplasms are well-described but considerably rarer gastric epithelial malignancies [14].

Two of the most widely accepted GAC classifications are the WHO and Laurén’s classifications.

The 2019 WHO classification recognizes five main subtypes of GAC: (i) tubular adenocarcinoma, (ii) papillary adenocarcinoma, (iii) mucinous adenocarcinoma, (iv) poorly cohesive carcinoma (PCC) and (v) mixed adenocarcinoma. GAC with lymphoid stroma, hepatoid carcinoma, micropapillary carcinoma and GAC of fundic-gland type represent rarer variants of GAC [14].

Laurén’s classification subdivides GAC into intestinal (53%), diffuse (33%) and indeterminate (14%) types [15]. This classification is important not only from a historical point of view but also from a methodological one. As described below, studies that investigate molecular alterations in GC are usually based on this classification. Moreover, Laurén’s two main types also present different biological characteristics: intestinal type GAC (IGAC) is usually sporadic and associated with Hp and Correa’s cascade [9], while diffuse GAC carcinoma (DGC) is characterized by loss of E-cadherin expression [16].

Two NGS-analysis-based molecular classifications of GC are commonly recognized: the 2014 Cancer Genome Atlas (TCGA) and the 2015 Asian Cancer Research Group (ACRG) classifications. TCGA classification distinguishes four molecular subtypes of GAC: (i) tumors positive for Epstein–Barr virus (EBV) (9%), (ii) microsatellite instability—high (MSI) tumors (21%), (iii) genomically stable (GS) tumors (20%) and (iv) tumors with chromosomal instability (CIN) (50%) [17]. According to ACRG results, GAC may be classified into four other subtypes: (i) microsatellite unstable (MSI) tumors (23%), (ii) microsatellite stable with features of epithelial–mesenchymal transition (MSS/EMT) tumors (15%), (iii) microsatellite stable with TP53 active (MSS/TP53+) tumors (26%) and (iv) microsatellite stable with TP53 inactive (MSS/TP53−) tumors (36%) [18].

The principal clinicopathological and molecular characteristics of TCGA and ACRG molecular subtypes are summarized in Table 1 and Table 2.

Molecular characterization of GC has provided valuable knowledge about the biology of this disease, permitting the development of targeted therapies (Table 3) [19].

## 2. Precursor Lesions of Intestinal Type Adenocarcinoma and Their Molecular Alterations

**Figure 1:**
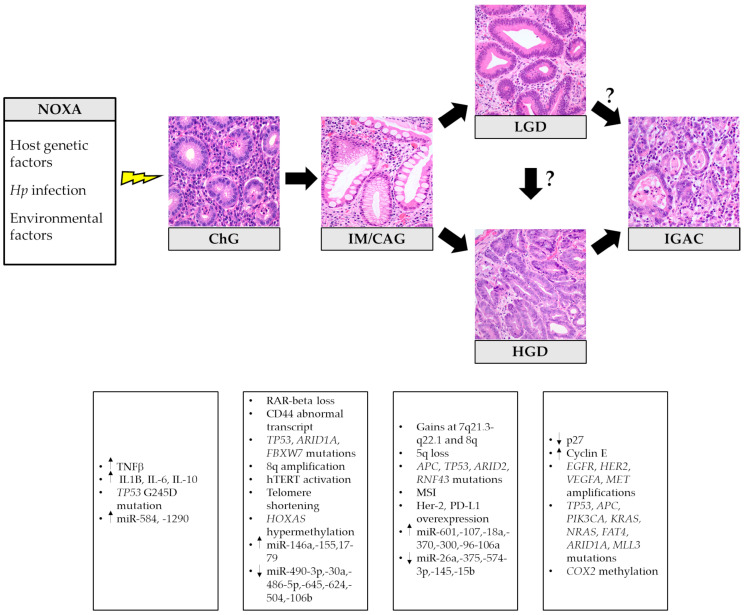
Histopathological stages and related principal molecular alterations in intestinal-type gastric cancer oncogenesis. Hp: *Helicobacter pylori*; ChG: chronic gastritis; IM/GAC: intestinal metaplasia/chronic atrophic gastritis; LGD: low-grade dysplasia; HGD: high-grade dysplasia; IGAC: intestinal-type gastric adenocarcinoma. The histological pictures were taken from routine diagnostic cases and have not been previously used for any other publication.

In 1975, Pelayo Correa first proposed a stepwise model of gastric carcinogenesis, which has revolutionized the comprehension of IGAC pathogenesis, allowing the introduction of effective GC prevention measures [32]. The so-called Correa’s cascade begins with the onset of chronic gastritis (ChG), possibly evolving into atrophic gastritis (i.e., loss of appropriate glands) [33] with the development of intestinal metaplasia (IM). Progressive acquisition of DNA mutations and molecular alterations leads to epithelial dysplasia. When neoplastic cells acquire the ability to invade the surrounding stroma, the final step of invasive carcinoma is reached (Figure 1) [34].

### 2.1. Chronic and Atrophic Gastritis

Hp is one of the main and most extensively studied etiological agents of ChG. Hp induces chronic gastric inflammation with atrophy, through the synthesis of molecules such as neutrophil myeloperoxidase–hypochlorite–hydrogen peroxide, macrophage nitric oxide and epithelial cell hydrogen peroxide. Consequent production of reactive nitrogen and oxygen species, lipid peroxidation, free radical formation and mutagens such as 8-oxo-7,8-dihydro-2′-deoxyguanosine and 8-nitroguanine induces cellular, DNA and RNA damage [35,36]. Additionally, Hp’s toxins (primarily CagA) interact with host proteins (e.g., ASPP2, RUNX3, PI3K, SHP2 and E-cadherin), leading to alterations in PI3K–AKT, Ras–ERK and Wnt pathways, inactivation of p53 and RUNX3, disruption of adherens junctions and deregulation of MGMT, MLH1 and CDKN2A [7,37].

Whole-exome sequencing and deep-sequencing analysis have shown the presence of somatic mutations in Hp-infected gastric samples, most notably non-synonymous low-abundance mutations in *TP53* and *ARID1A*. In particular, TP53 G245D is the most frequent reported mutation in Hp gastritis [38]. Mutations in Hp gastritis are predominantly C:G > T:A transitions in GpCpX sequences, which are a marker of activation-induced cytidine deaminase (AID) activity and a typical molecular signature of GC [38,39]. Moreover, CagA toxin also upregulates some microRNAs (miRNAs) such as miRNA-584 and miRNA-1290 [40]. 

Within the context of post-transcriptional regulation, several miRNAs and long non-coding RNAs (lcnRNAs) have been found to be differentially expressed in Hp-related ChG, when compared to normal mucosa or GC [41,42,43]. For example, Hp-infection-related lncRNA AF147447 has been reported to act as a tumor suppressor and to be dysregulated during the multistep cascade of gastric carcinogenesis [44].

The final effects of these processes are cellular proliferation, DNA hypermethylation, loss of apoptotic capacity and epithelial–mesenchymal transition [7]. The acquisition of aforementioned molecular alterations is morphologically unveiled by the development of IM, low/high grade dysplasia and IGAC in the setting of Hp gastritis.

Hp is undoubtedly the major risk factor for GC. However, it is not the only microbial agent involved in gastric carcinogenesis. By performing 16S rRNA gene analysis, differences in microbial diversity and richness between GC and atrophic gastritis and IM were found, indicating the role of microbial dysbiosis, especially of oral bacteria, in gastric carcinogenesis [45,46]. Furthermore, specific clusters of oral bacteria were found to be depleted following Hp eradication in patients with persistent inflammation [47].

Because Hp-induced inflammation is the most important mechanism of gastric carcinogenesis, the role of the immune microenvironment in GC progression is of great interest but is yet to be elucidated. Neutrophil density is positively correlated with gastric epithelium cell proliferation in gastritis, suggesting neutrophils might promote cell proliferation [48]. Furthermore, CCL5+ T cells, which are presumably activated cytotoxic T cells, together with neutrophils, seem to play an important role in the active inflammatory process of ChG [49].

Autoimmune gastritis (AIG) is another important cause of ChG. Despite being considered responsible for less than 5% of gastritis, AIG is often underdiagnosed, and its epidemiological burden is probably higher [50]. In AIG, the adaptive immune system selectively damages oxyntic cells, leading to corpus/fundus atrophy eventually with hypo- and achlorhydria. In the context of AIG, IM and spasmolytic polypeptide-expressing metaplasia (SPEM) may also develop. Following Correa’s cascade, dysplasia and invasive carcinoma may subsequently occur. While the exact risk of GC in AIG is still undefined [50,51,52], some authors suggest that GC in AIG is mostly confined to patients with concomitant Hp infection [53]. It is worth remembering that AIG is also associated with an increased risk of neuroendocrine tumors (NETs). Oxyntic-atrophy-induced hypergastrinemia induces hyperplasia of enterochromaffin-like cells, which may progress to dysplasia and development of type I NETs [50].

In chronic gastritis, different cytokines (IL-8, TNF-alpha, IL-10, IL-1B, IL-1RA, IL-22, IL-23, IL-32, IL-33) seem to have a role in gastric carcinogenesis, tumor growth, metastatic potential and/or chemoresistance [54]. Moreover, IL-1beta has been reported to induce aberrant DNA methylation in a mouse model [55]. These observations suggest that GC risk may also be influenced by the type of cytokines produced by different clusters of T helper lymphocytes responding to Hp or H+/K+ ATPase antigens.

### 2.2. Intestinal Metaplasia and the “Point of No Return”

IM is defined by the replacement of native gastric mucosa by an epithelium with intestinal commitment. IM is associated with an estimated GC risk of 0.13–0.25% per year [56]. Median time of progression to GC in patients with IM is reported to be 6.1 years [57]. Three main IM subtypes are recognized [58,59]. The most common is type I IM (complete or small intestinal type), characterized by mature enterocytes admixed with Paneth cells and sialomucins producing goblet cells, with a considerably decreased expression of gastric mucins (MUC1, MUC5AC and MUC6) in favor of intestinal mucin (MUC2). Type II (incomplete or immature, colonic type) presents neutral/acid sialomucins secreting columnar cells in various stages of differentiation and goblet cells usually secreting sialomucins, together with a lack of absorptive cells. Type III (incomplete) is defined by the presence of columnar cells secreting acid sulfomucins. Both type II and III express MUC1, MUC5AC and MUC6. Differences in commitment between complete and incomplete IM are also highlighted by the different expression of gastric (SOX2) and intestinal (CDX2) transcription factors: while CDX2 is expressed in all types of IM, SOX2 is negative in 93% of complete IM and positive in 85% of incomplete types [60]. Despite the fact that some authors [61,62,63,64,65,66,67,68,69,70,71,72,73,74] suggest that incomplete IM is associated with increased risk of GC, there is no conclusive evidence of differences in cancer progression among IM types, and IM characterization is not currently recommended in clinical practice [59].

A mutation rate of 2.6 mutation/Mb has been reported in IM. This value is higher than the normal gastric mucosa mutation rate (0.4 mutations/Mb) but lower than that reported in non-hypermutated GC (6.9 mutations/Mb) [65]. Metaplastic regions taken from the same patient seem to be genetically distinct and IM is probably a condition with high intra-patient multiclonality. Mutations in IM are frequently represented by C > T transitions in CpG dinucleotides, a mutational signature that correlates with age at cancer diagnosis. T > G transitions, a signature putatively associated with gastric acid, have also been described in IM [65]. *TP53* and *ARID1A* are two of most frequently mutated genes in GC. Interestingly, in the series of Huang and colleagues [65], *TP53* and *ARID1A* are reported to be mutated only in 2% and 3% of IM samples. Other authors describe p53 alterations and *TP53* loss of heterozygosis, respectively in 30% and 14% of IM regions [66,67].

In the Huang series [65], *FBXW7* is the only significantly mutated gene in IM identified by MutSigCV analysis. Despite being a driver gene, *FBXW7* is mutated only in 4.7% of IM versus 9.2–18.5% of GC. Comparing these different values, it is clear that additional genomic alterations are needed for the development of GC [65]. Mutations of *APC* have also been reported in IM [59]. 

Somatic copy number alterations (sCNAs) have been described in 12.5% cases of IM, the most common being chromosome 8q (8q22.3–8q24.3) amplification [65]. Notably, this amplified locus also contains the *MYC* oncogene (8q24.21). IM presents shorter telomere length when compared to normal gastric mucosa. In particular, antral IM has shorter telomeres than corpus/cardiac IM [65]. An association between 8q amplification and telomere shortening has been suggested [65]. Interestingly, telomere length seems to be similar between GC and normal gastric mucosa, but advanced GC has longer telomeres than early GC [65,68]. Considering these results, it is possible to speculate that telomeres are initially eroded in IM and subsequently restored during progression to advanced GC.

DNA methylation levels are higher in IM than in mucosa with ChG [64]. Antral IM presents higher levels of DNA methylation than corpus/cardiac IM. Moreover, high levels of somatic mutations and sCNAs have been associated with highly methylated antral IM [65]. In contrast, global intragenic hypomethylation has not been detected in IM, representing a later event in Correa’s cascade. Finally, Lin and colleagues [69] suggest that hypermethylation of *HOXA5* has a role in the development of gastric cardiac IM.

Other molecular alterations occurring in IM are loss of RARbeta, production of CD44 abnormal transcripts and microsatellite instability [9]. Microsatellite instability has been documented in incomplete IM adjacent to MSI GC, suggesting that it may be an early event in gastric carcinogenesis [70].

IM seems to be related also to dysregulation in miRNAs’ pathways. Levels of miRNA-146a and miRNA-155 are higher in patients with Hp-induced IM than in healthy subjects [71]. According to Li and colleagues [72], the miRNA 17-92 cluster (i.e., miRNA-17-5p, -17-3p, -20a, -18a, -92a, -19a and -19b) is upregulated in IM and could be possibly used as a serum biomarker for early detection of IM and GC. Reported downregulated miRNAs in IM are miRNA-490-3p and miRNA-30a [73,74,75]. Deregulation of hsa-miRNA-486-5p, -645, -624, -504 and -106b has also been reported in IM [76].

Even though Correa’s cascade is described as a linear process, gastric carcinogenesis appears to be rather more dynamic. Pre-neoplastic lesions can regress or evolve, and progression may even abruptly skip some theoretical stages. The efficacy of Hp eradication in inducing regression of ChG/AG and in reducing the risk of GC progression is well documented [64]. The so-called “point of no return” represents a stage in gastric carcinogenesis in which Hp eradication leads to no substantial advantages in terms of histological regression and GC risk reduction. Different studies have suggested that the point of no return may be the IM stage [77,78,79,80,81,82]; however, regression of IM to ChG/AG has also been reported [62,83]. Taken together, these results suggest that IM is a condition with low probability of regression after Hp eradication. The irreversible molecular alterations inducing the point of no return remain unknown.

Risk of GC progression in ChG/AG is routinely assessed using the OLGA and OLGIM histological classifications [84,85]. However, molecular factors associated with GC progression in patients with IM still need to be characterized. According to Huang [65], there is a positive association between high levels of DNA methylation and IM progression. The presence of sCNAs and shortened telomeres are other molecular features associated with IM progression [65].

### 2.3. Spasmolytic Polypeptide-Expressing Metaplasia

Also known as pseudo-pyloric metaplasia, SPEM is a metaplastic process occurring in the gastric corpus and fundus characterized by morphologic and phenotypic features of antral glands without the G-cell component [86]. Strong expression of trefoil factor 2 (TFF2) and MUC6 are typically seen in SPEM [59]. Many studies have associated SPEM with GC, and it has been speculated that SPEM may play a role in GC carcinogenesis [87,88,89]. However, SPEM is not a defined step in Correa’s cascade, and there is no agreement in considering SPEM a defined precursor lesion of IGAC and not a mere associated phenomenon [86,90]. Of note, results from studies on mouse models and human metaplastic tissues indicated that M2 macrophages may act as promoters of the advancement of SPEM in the presence of inflammation [91].

### 2.4. Dysplasia

Formerly known as intraepithelial neoplasia (IEN) or non-invasive neoplasia (NiN), gastric dysplasia is currently defined as unequivocal neoplastic changes of gastric epithelium without evidence of stromal invasion [14]. Low-grade dysplasia (LGD) presents minimal architectural disarray with mild to moderate cytologic atypia and mitotic activity. High-grade dysplasia (HGD) is characterized by cuboidal/columnar cells with marked cytologic atypia, brisk mitotic activity, high nucleus/cytoplasm ratio and prominent nucleoli together with a complex glandular architecture [14]. Risk of GC progression substantially increases from LGD (4–18%) to HGD (up to 69%) [92]. Moreover, within a year from initial diagnosis, patients with LGD and HGD have a risk of GC development, respectively, of 2.1% and 24.9% [93].

Analysis of low- and high-grade gastric dysplastic lesions displays a mean number of genetic regions with high-level sCNAs of 0.2 [94]. The frequency of sCNAs is higher in HGD than in LGD [95]. In the series of Uchida and colleagues [94], the most frequent sCNAs in gastric dysplasia were gains at 7q21.3–q22.1 (55%), 8q (40%) and 7pq (35%), and loss at 5q (30%). In particular, gains at 7pq and 8q have been, respectively, reported in 54% and 62% of high-grade dysplasia but not in low-grade dysplasia. On the other hand, low-grade cases had more gains at 1q, 17q, 21q and 22q than high-grade dysplasia, while gains in 7q21.3–q22-1 and loss of 5q presented similar frequencies in the two groups. 

A median mutation density of 11.3 mutations/Mb has been reported in gastric dysplasia, with C > T transition being the most common mutation type, followed by C > A and T > A [82]. Rokutan and colleagues [92] reported *APC* as the most frequently mutated gene in gastric dysplasia (76% of cases). According to their results, all LGDs and about a half of HGDs harbored APC mutations, and the co-occurrence of *APC* and *ARID2* mutations was a common event. Despite the different mutational rate, *APC* mutations present identical hotspots in both LGD and HGD. This observation could support the hypothesis that APC-mutated LGD and HGD are biologically related, with the latter being a progression of the former [92]. *APC* or *TP53* mutations have been reported in about 88% of gastric dysplasia, with *TP53* mutated exclusively in HGD. Interestingly, the risk of cancer progression seems to be higher in *TP53*-mutated gastric dysplasia than in *APC*-mutated ones [92,96]. This hypothesis is supported by the well-documented role of *TP53* in GC progression [67,97]. Finally, mutations in *ARID2*, *MUC6*, *TP53*, *KRAS*, *BRAF*, *PIK3CA* and *FBXW7* have also been described in both LGD and HGD [92,95].

*TP53* is the most frequently mutated gene in HGD [67]. Other HGD-related mutations are described in *APC*, *ATM*, *STK11*, *PIK3CA*, *RB1*, *CDKN2A*, *FGFR3*, *IDH2*, *MET*, *RNF43*, *RET* and *G0S2* [14,67,93]. Since most of these mutations have also been reported in invasive GC, they probably play an early role in gastric carcinogenesis [67]. In comparison to normal gastric mucosa, both LGD and HGD also present downregulation of *BCL2L11*, *RET* and *ALB* and overexpression of *AEG*-1, *GRIN2D* and *BRCA1* [98,99].

The expression of druggable molecules has also been reported in gastric dysplasia. *HER2* overexpression and *HER2* amplification have been described in both LGD and HGD, being more frequent in the latter [100]. According to these results, *HER2* dysregulation seems to be an early event in gastric carcinogenesis.

PD-L1 expression has also been documented in gastric dysplastic lesions, and it has been related to mismatch repair deficiency (MMRd) [101].

In comparison to normal mucosa, gastric dysplastic lesions and early gastric cancer (EGC) present downregulation of miRNA-26a, -375, -574-3p, -145 and -15b together with upregulation of miRNA-601, -107, 18a, -370, -300 and -96 [102]. Zhu and colleagues [103] report a gradual increase in miRNA-106a expression from LGD to HGD and EGC. Finally, dysregulation of miR-125a-5p/125b is an early event in gastric intestinal-type carcinogenesis. Interestingly, miR-125 expression has been inversely related to Her2 status, representing a possible therapeutic target in Her2-positive GC [104].

As regards epigenetic changes, Hp has been found to enhance histone H3 serine 10 phosphorylation within the progression from ChG to IM and finally to dysplasia. On the contrary, Hp is responsible for promoting gastric carcinogenesis via downregulation of the activity of many histone deacetylases (HDACs) (i.e., HDAC6, SIRT1) [105,106,107].

Understanding the molecular biology of gastric dysplastic lesions is a fascinating field and, as shown above, different studies have provided great knowledge on this matter. Despite having discovered many molecular alterations with a crucial role in gastric dysplasia development, a lack of comprehension concerning the biology of gastric dysplasia still remains. In particular, it is not clear whether LGD and HGD are strictly related, in a biological and molecular fashion. According to gene expression analysis of Xu and colleagues [93], there is a clear biological distinction between LGD and HGD. This hypothesis is also supported by other studies [64,92]. Fassan and colleagues [67] suggest that HGD is molecularly similar to EGC. Moreover, Hwang and colleagues [102] outline that gastric dysplastic lesions and EGC have different profiles of miRNA expression. In contrast, a recent study by Zhang and colleagues [98] reports that the gene expression profiles of LGD and HGD are more similar to each other than to that of EGC.

Another important factor significantly affecting a rapid comprehensive evaluation of the molecular background sustaining gastric dysplasia is the heterogeneous phenotypical landscape of gastric dysplasia itself. In fact, two main subtypes of gastric dysplasia have been described according to histological features and immunophenotyping: the intestinal and the gastric type. The gastric type can be further divided into the foveolar and pyloric types [4]. These lesions may occur de novo from the native gastric mucosa, outside the multistep GC carcinogenetic model, show features of biological aggressiveness and may represent the putative precursor lesion of gastric-type adenocarcinoma [108]. Mixed phenotypes are frequently observed in the clinical practice, and thus, most of the published molecular studies did not distinguish among the two major entities. Furthermore, most of the available data relate to intestinal-type dysplastic lesions. In a recent paper, Sugai and colleagues [109] investigated a relatively large series of intestinal-type and foveolar-type low-grade dysplastic lesions and found that foveolar-type dysplasia is characterized by a higher prevalence of allelic imbalance and a low methylation epigenotype.

### 2.5. Special Type of Gastric Adenomas

Besides the Correa’s multistep GC carcinogenetic cascade, four distinct special-type gastric adenomas are described in the 2019 WHO classification [14]: the intestinal-type adenoma, the foveolar-type adenoma, the gastric pyloric gland adenoma and oxyntic gland adenoma.

Intestinal-type gastric adenoma differs from gastric dysplasia because of its polypoid nature. It may develop in the context of diffuse atrophic gastritis; therefore, its genetic landscape is similar to what has been described for the multistep carcinogenetic process and somewhat analogous to what has been observed in colorectal adenomas. In particular, these lesions are characterized by mutations in the *APC*, *KRAS*, *ERBB2* and *ARID2* genes but not alterations in *CTNNB1* [110,111]. Some cases show a microsatellite instability phenotype. Syndromic cases typically occur as part of familial adenomatous polyposis.

Foveolar-type adenoma consists of a polypoid lesion covered by neoplastic foveolar epithelium that mostly occurs in an otherwise healthy oxyntic gastric compartment. Sporadic cases are rare, whereas most of the lesions are detected in the context of familial adenomatous polyposis and GAPPS [14]. Sporadic cases infrequently present mutations in the *APC* and *KRAS* genes [110].

Gastric pyloric gland adenomas are epithelial polyps consisting of neoplastic pyloric-type glands. They are usually associated with atrophic changes of the oxyntic mucosa, e.g., those due to autoimmune and/or Hp-related gastritis [112], but they may also be associated with FAP. Most gastric pyloric gland adenomas feature activating mutations in *GNAS* and *KRAS* and inactivating *APC* mutations [113,114].

Oxyntic gland adenomas are benign neoplasms of the upper third of the stomach composed of columnar cells with differentiation to chief cells, parietal cells or both, mostly occurring in a non-atrophic oxyntic mucosa background [14]. This adenoma subtype is characterized by a high rate of progression to GC of fundic gland type. A third of the cases show missense or non-sense mutations in the WNT/β-catenin pathway, except for *CTNNB1* [115].

## 3. Precursors of Diffuse Gastric Cancer

Diffuse gastric cancer (DGC) accounts for nearly 30% of GCs [15]. DGC is macroscopically characterized by a diffuse thickening of the gastric wall (i.e., *linitis plastica*) and usually presents a poorly cohesive histological phenotype. Loss of E-cadherin expression is the principal molecular feature of DGC [16]. DGC is usually sporadic, but familial clustering is reported in 10% of cases, and 1% to 3% arise in the context of a hereditary syndrome [116].

### 3.1. Hereditary Diffuse Gastric Cancer

HDGC accounts for 1–3% of gastric cancers and usually presents at a young age [116]. 

The cumulative incidence of invasive GC by the age of 80 years is 70% in men and 56% in women. Individuals with HDGC also bear an increased risk of lobular breast carcinoma and possibly of colorectal carcinoma [117].

Germline mutations of *CDH1* are the genetic basis of HDGC in up to 40% of cases [118]. To date, at least 100 pathological *CDH1* germline mutations have been reported [119]. Small insertions and deletions represent the most common mutations (35% of cases), followed by large exon deletions and missense mutations (28%), non-sense mutations (16%) and splice site mutations (16%) [120]. 

Mutations of *CTNNA1*, *PALB2*, *MYD88* and *PIK3CA* have also been related to HDGC [121]. Interestingly, HDGC patients with germline CDH1 mutations have lower survival rates than those with non-CDH1 germline mutations [122].

Based on the analysis of prophylactic gastrectomy of HDGC patients with *CDH1* germline mutation, the Carneiro model proposes a carcinogenetic pathway to DGC [123]. According to this paradigm, the pivotal (but not obligatory [124]) precursor lesion is represented by signet ring cell carcinoma (SRCC) in situ. Histologically, in situ SRCCs are composed by signet ring cells with eccentric hyperchromatic nuclei and mucinous vacuoles confined in glands with an intact basement membrane [123]. A pagetoid spread of signet ring cells below normal foveolar epithelium is frequently recognized. Loss of E-cadherin expression is an early event, as it is already documented at the stage of in situ SRCC and subtending the phenotypic transition from normal gastric mucosa to pre-invasive lesion [123]. E-cadherin loss is induced by an alteration of the second *CDH1* allele [125]. About 50% of those second-hit inactivations is represented by *CDH1* promoter hypermethylation [126]. Other less frequent mechanisms are *CDH1* mutations or loss of heterozygosity [127].

Complete loss of *CDH1* expression allows neoplastic cells to detach from the basement membrane, but other gene alterations are needed for the progression to invasive carcinoma [117]. For example, a strong expression of C-Src kinase (involved in epithelial–mesenchymal transition) has been documented in neoplastic cells invading the muscularis mucosae but not in intramucosal signet ring cells [128]. Further studies are required to better comprehend the molecular biology of HDGC and to understand genetic and epigenetic alterations involved in DGC oncogenesis.

### 3.2. Sporadic Diffuse Gastric Cancer

Up to 70% of sporadic diffuse gastric cancers (SDGCs) harbor somatic mutations in CDH1 [59]. Moreover, somatic mutations of TTN have been reported in 40% of cases [129]. In contrast to HDGC, SDGC is strongly related to Hp infection [130,131]. Chan and colleagues [132] suggest that Hp may induce *CDH1* promoter methylation in non-neoplastic gastric mucosa and in gastric cancer. Hp eradication may lead to the reversion of methylation in non-neoplastic gastric mucosa [133].

A further clue that HDGC and SDGC may have different pathogenesis is provided by Lee and colleagues [134]. According to their results, HDGC cases are negative for CDX2, while SDGCs present CDX2 positivity. Moreover, a significant association between IM and SDGC development has been described [135], and OLGA/OLGIM stages III/IV have been associated not only with IGAC but also with DGC [136]. All together, these data suggest that SDGCs may develop from an “alternative” carcinogenetic pathway in the context of atrophic/metaplastic gastritis in which CDH1 mutations play a pivotal role.

## 4. Conclusions

GC still represents an aggressive and often deadly neoplasm. Despite great efforts to develop effective treatments, GC overall survival is unacceptably poor. Looking back on the recent past, two great successes in the fight against GC have been achieved thanks to a better comprehension of GC pathogenesis. The discovery of the role of Hp in GC has allowed the medical community to understand the importance of Hp eradication, with great benefits in terms of GC incidence and mortality reduction. Moreover, Correa’s cascade has highlighted the multiphasic nature of intestinal-type gastric adenocarcinoma, permitting the development of effective programs of primary and secondary prevention. It is tempting to say that understanding the molecular landscape of pre-invasive gastric lesions could lead to the next milestone discovery in GC prevention. The rapidly accumulating data about the molecular alterations in GC carcinogenesis partially support this hope. The importance of methylation patterns and chromosome 8 status in IM evolution or *TP53* mutational status in gastric dysplasia progression is not negligible: these data could be useful in improving the risk-assessment algorithms. Our ability to diagnose GC and/or gastric pre-invasive lesions could be improved by the use of molecular biomarkers, such as miRNAs. Moreover, the expression of druggable molecules such as Her2 and PD-L1 in gastric dysplasia should be taken into consideration in selecting patients for targeted therapeutic strategies. However, further efforts should be made to shed light on some obscure points. For example, the “point of no return” in gastric carcinogenesis remains elusive. Moreover, comprehensive knowledge about the biology of LGD and HGD and their relationship is still lacking. What is clear is that, in the era of precision oncology, a detailed molecular characterization of GC carcinogenesis represents a formidable tool to optimize the diagnostic and therapeutic decision-making process.

## Figures and Tables

**Table 1:** Principal molecular and clinicopathological features of TCGA molecular subtypes.

TCGA			
CIN (50%)	EBV (9%)	MSI (21%)	GS (20%)
GEJ/cardia Intestinal type DNA aneuploidy Highly variable CIN *TP53* mutations Amplification of TKR	Male prevalence Gastric corpus or fundus Extensive DNA promoter methylation *CDKN2A* promoter hypermethylation *PIK3CA*, *ARID1A* and *BCOR* mutations	Elderly age Gastric antrum Intestinal type Best prognosis among TCGA subtypes *MLH1* promoter hypermethylation High mutational burden Possibly associated with Lynch syndrome	Younger age Distal localization Poorly cohesive histotype Worst prognosis among TCGA subtypes Low CNAs and mutational burden *ARID1*, *RHOA* and *CDH1* mutations *CLDN18–ARHGAP26* fusion in 15%

**Table 2:** Principal molecular and clinicopathological features of ACRG molecular subtypes.

ACRG			
MSS/TP53− (36%)	MSS/TP53+ (26%)	MSI (23%)	MMS/EMT (15%)
Male predominance Intestinal type Highest rate of *TP53* and *RHOA* mutations *APC*, *ARID1A*, *KRAS*, *PIK3CA* and *SMAD4* mutations	Male predominance Intestinal type EBV infection *ARID1A*, *PIK3CA*, *SMAD4* and *APC* mutations	Gastric antrum Intestinal type Early stage at diagnosis Best prognosis among ACRG subtypes DNA methylation signature Presence of hypermutation Silencing of *MLH1* *ARID1A*, *KRAS* and *ALK* mutations PIK3–PTEN–mTOR pathway dysregulation PD-L1 overexpression	Younger age Poorly cohesive histotype Higher frequency of peritoneal spreading Worst prognosis among ACRG subtypes Loss of *CDH1* expression

**Table 3:** Principal and promising targeted and immunotherapies in advanced gastric cancer.

MOLECULAR TARGET	THERAPEUTIC AGENT (Trial)	LINE OF THERAPY
HER2	Trastuzumab (ToGa [20]) Trastuzumab deruxtecan (DESTINY-Gastric 01 [21])	First line Third or later line
FGFR2	Bemarituzumab (FIGHT [22,23])	First line
MET	Onartuzumab (METGastric [24]) Savolitinib (VIKTORY [25])	Second line
VEGF/VEGFR	Ramucirumab (RAINBOW [26]) (REGARD [27])	Second line Second line
CLAUDIN 18.2	Zolbetuximab (FAST [28])	First line
PD-1/PD-L1	Nivolumab (CHECKMATE-649 [29]) (ATTRACTION-4 [30]) (ATTRACTION-2 [31])	First line First line Third or later line

## Data Availability

Data available upon request.
